# Educating Outpatients for Bowel Preparation Before Colonoscopy Using Conventional Methods vs Virtual Reality Videos Plus Conventional Methods: A Randomized Clinical Trial

**DOI:** 10.1001/jamanetworkopen.2021.35576

**Published:** 2021-11-22

**Authors:** Guorong Chen, Yi Zhao, Feng Xie, Wen Shi, Yingyun Yang, Aiming Yang, Dong Wu

**Affiliations:** 1 Department of Gastroenterology, State Key Laboratory of Complex Severe and Rare Diseases, Peking Union Medical College Hospital, Chinese Academy of Medical Sciences, Beijing, China; 2 Department of Gastrointestinal Surgery, Peking University Cancer Hospital, Beijing, China; 3 Department of Health Research Methods, Evidence and Impact, McMaster University, Hamilton, Canada; 4 Centre for Health Economics and Policy Analysis, McMaster University, Hamilton, Canada

## Abstract

**Importance:**

Adequate bowel preparation is essential for diagnostic, screening, and surveillance colonoscopy. Virtual reality (VR) has the characteristics of immersion, interaction, and imagination and has been widely used in medicine for training and teaching, indicating that it could be used in the education of outpatients for bowel preparation before colonoscopy.

**Objective:**

To investigate whether using VR videos for patient education before colonoscopy could improve bowel preparation.

**Design, Setting, and Participants:**

A prospective, single-blinded, randomized clinical trial of 346 patients undergoing colonoscopy with local anesthesia in a tertiary care hospital was conducted between October 1, 2018, and November 1, 2020. Outpatients who had indications for colonoscopy and had not received one before were enrolled. Statistical analysis was performed from November 1 to December 31, 2020. All data were analyzed according to the intention-to-treat approach.

**Exposures:**

Conventional bowel preparation education (oral instructions and written materials that had the same contents) or conventional education plus VR videos.

**Main Outcomes and Measures:**

The primary outcome was the quality of bowel preparation measured by the Boston Bowel Preparation Scale score (range, 0-9, where 0 indicates extremely unsatisfactory bowel preparation and 9 indicates complete bowel preparation). Secondary outcomes included polyp and adenoma detection rates, compliance with complete bowel cleansing, preprocedure anxiety, overall satisfaction, and willingness to undergo a follow-up colonoscopy.

**Results:**

A total of 346 outpatients were enrolled in the trial, with 173 patients randomly assigned to each group (control group: 87 women [50.3%]; mean [SD] age, 50.5 [12.5] years; VR video group: 84 women [48.6%]; mean [SD] age, 52.6 [11.4] years). Baseline characteristics, including demographic information, medical history, lifestyle, and the characteristics of stool, were comparable between the VR video group and the control group. The mean (SD) Boston Bowel Preparation Scale score was significantly higher in the VR video group than in the control group (7.61 [1.65] vs 7.04 [1.70]; *P* = .002). The detection rate of polyps (72 of 172 [41.9%] vs 46 of 172 [26.7%]; *P* = .003) and the detection rate of adenomas (56 of 172 [32.6%] vs 38 of 172 [22.1%]; *P* = .03) were also higher in the VR video group. Patients who received VR education had better compliance (119 [68.8%] vs 87 [50.3%]; *P* < .001) and higher mean (SD) overall satisfaction (8.68 [1.70] vs 8.16 [2.15]; *P* = .01) with bowel preparation.

**Conclusions and Relevance:**

Patients who received VR video education before colonoscopy had better bowel preparation, higher polyp and adenoma detection rates, and improved compliance and satisfaction.

**Trial Registration:**

ClinicalTrials.gov Identifier: NCT03667911

## Key Points

**Question:**

Can educational virtual reality videos for patients improve the quality of bowel preparation before colonoscopy?

**Finding:**

This randomized clinical trial including 346 participants found that patients educated by virtual reality videos had a higher mean Boston Bowel Preparation Scale score, indicating better bowel preparation, than patients educated by conventional methods.

**Meaning:**

Virtual reality videos can be used as an effective patient education tool for bowel preparation before colonoscopy.

## Teaser

This randomized clinical trial investigates whether using virtual reality videos for patient education before colonoscopy could improve bowel preparation.

## Introduction

Colonoscopy is the standard method to screen for and detect colorectal adenoma and carcinoma among the general population and those at high risk.^1^ The extent of bowel cleansing is significantly associated with the detection rate of colorectal adenoma.^2,3,4^ Optimal bowel preparation is essential for endoscopists to observe intestinal mucosa clearly, and inadequate bowel preparation increases the difficulties in procedure, interferes with the endoscopist’s judgment, and decreases the detection of lesions.^5^

Inadequate bowel preparation often results from poor patient compliance. Mahmood et al^6^ concluded that age, sex, educational level, personal preference, and income status were associated with the quality of bowel preparation. This finding highlights the importance of educating and motivating patients to improve compliance and obtain better bowel cleansing.^7,8^ Apart from conventional oral and written instructions, multiple studies have shown that providing education via the telephone, smartphone applications, or educational videos improved the quality of bowel preparation and, as a result, led to a higher detection rate of adenoma.^9,10,11,12,13^

Virtual reality (VR) is used widely in education and entertainment for its immersion, interaction, and imaginative characteristics. Hann et al^14^ showed that VR could be conveniently incorporated into daily endoscopic work to improve assessment of lesions, such as polyps. Virtual reality simulation–based training has also been used to supplement conventional endoscopy training.^15^ Some physicians can improve their endoscopic skills with VR training, and we postulate that patients may also benefit from VR education to improve their motivation for and experience of endoscopic procedures. For that purpose, we invented a head-mounted, 3-dimensional display device to educate patients about bowel cleansing and the colonoscopic procedure using scenes from an actual video-recorded colonoscopy. We hypothesized that VR videos could improve bowel preparation by enhancing patient’s motivation, reducing preprocedure anxiety, and improving patient’s experience during colonoscopy under local anesthesia.

## Methods

### Study Design

**Figure 1.**
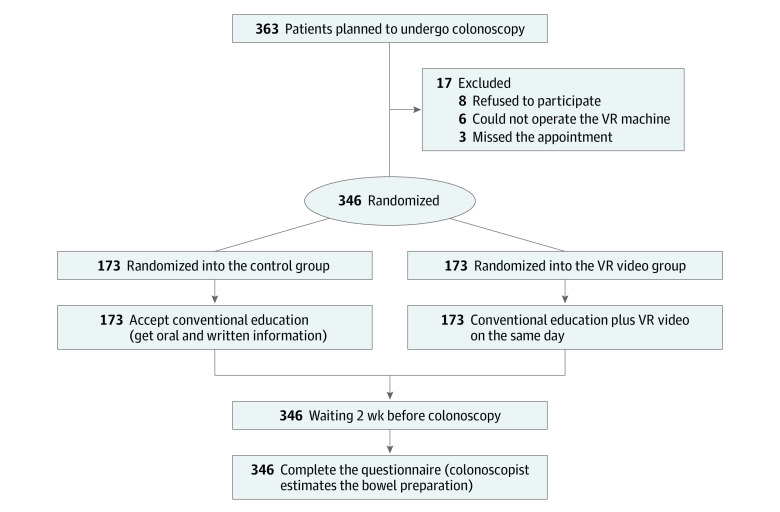
Flowchart of the Study VR indicates virtual reality.

We followed the Consolidated Standards of Reporting Trials (CONSORT) reporting guideline in reporting this trial. The trial protocol was published elsewhere^16^ and is available in Supplement 1. In brief, the study was a prospective, single-blinded, randomized, single-center clinical trial conducted at a tertiary care hospital in Beijing, China, between October 1, 2018, and November 1, 2020. Outpatients scheduled for screening or diagnostic colonoscopy under local anesthesia for the first time were enrolled consecutively and randomly assigned to the control group or the VR video group according to a random number table (Figure 1). The institutional review board and ethics committee of the Peking Union Medical College Hospital approved this study. All patients signed written informed consent.

### Patients

Outpatients aged 18 to 75 years who had indications for a colonoscopic examination for screening or diagnostic purposes and who had not received a colonoscopy before were enrolled. All patients presented to Peking Union Medical College Hospital, a tertiary hospital in Beijing, China. Exclusion criteria included the following: (1) contradictions for colonoscopy; (2) history of bowel surgery, especially colorectal surgery; (3) severe comorbidities (eg, ascites, congestive heart failure, chronic renal failure, and coronary artery disease within the last 6 months); (4) constipation (typically <3 bowel movements per week with hard stools, a feeling of incomplete evacuation, or abdominal discomfort) requiring the use of laxatives; (5) pregnancy or lactation; (6) diagnosis of inflammatory bowel disease; and (7) unable to watch VR videos (eg, owing to blindness).

After providing written informed consent, patients set up an appointment for a colonoscopy 2 weeks after. They were randomized into 2 groups with a ratio of 1:1 (the conventional education group or the conventional education plus VR video group [hereafter referred to as the VR video group]). We used a computerized random number table to achieve randomization. A physician (Y.Y.) who was not involved in the colonoscopy performed the randomization and allocated patients into the 2 groups. All examinations were performed in the morning, and all colonoscopic procedures were performed by 1 endoscopist (D.W.) who was unaware of the allocation.

### Education on Bowel Preparation

All patients received bowel preparation instructions once their appointments were confirmed. In this trial, all of the patients’ waiting times between making the appointment and undergoing colonoscopy were approximately 2 weeks’ long. A well-trained physcian (G.C. or Y.Z.) provided an in-person educational session about colonoscopy, including the importance of bowel preparation, the possible adverse effects of the laxative, and other instructions. In addition, all patients received a written instruction containing the following details concerning (1) dietary requirements in which the patients need to restrict their diet (low-residue diet, avoiding vegetables, fruits, cereal, and fried foods) starting at least 1 day before their appointment and (2) colon cleansing regimens in which all patients would receive the same dose of laxatives (polyethylene glycol) and take the first 2 L of laxatives on the evening of the day before the colonoscopy (after dinner). The second dose (1 L of laxatives) needs to be administered 3 to 4 hours before the colonoscopy (eFigure in Supplement 2).

For the control group, patients received only conventional education on bowel preparation before colonoscopy. Written materials that had the same contents as the oral instructions were also provided.

**Figure 2.**
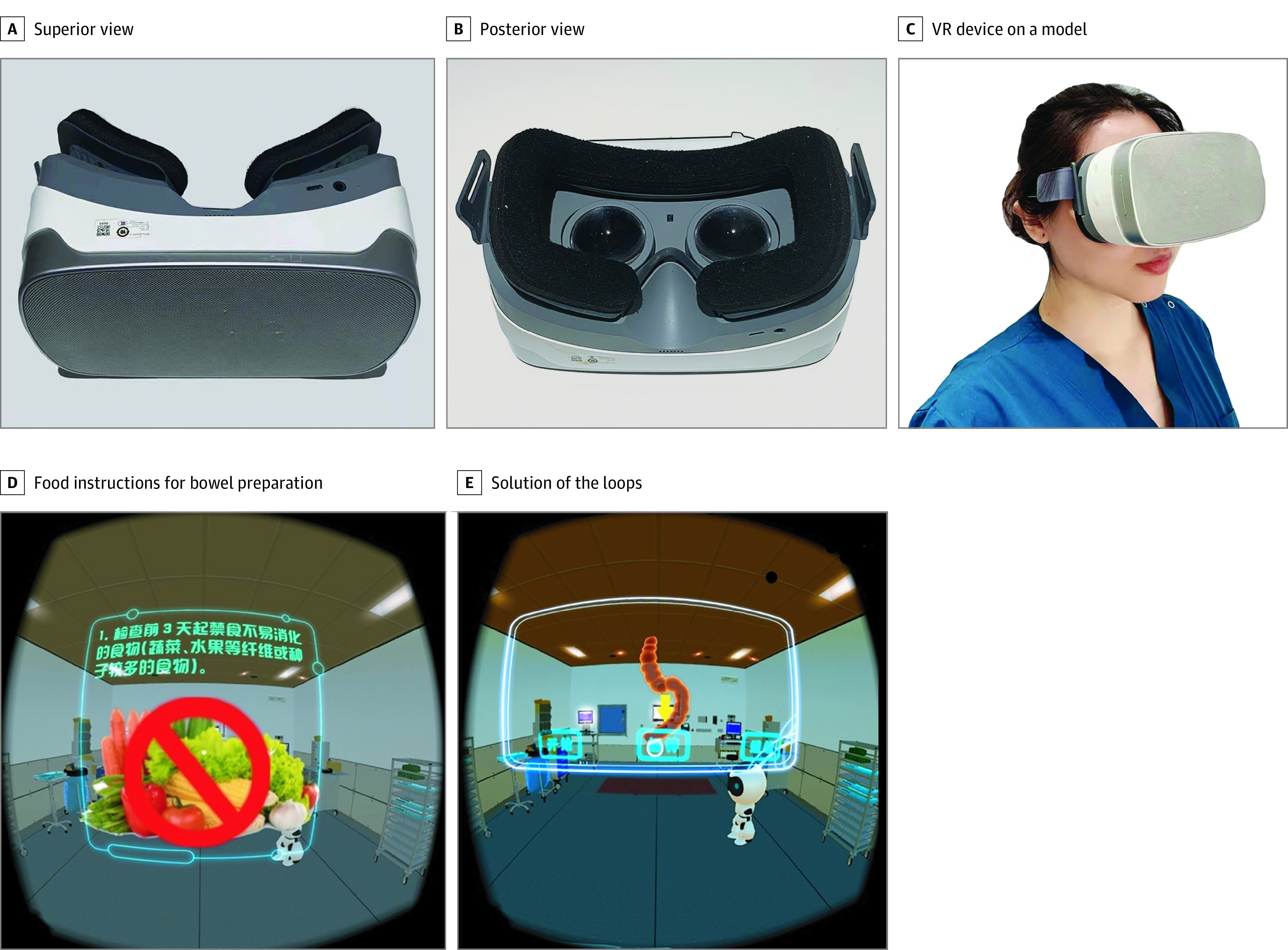
Head-Mounted Display for Virtual Reality (VR) Videos

For the intervention group (conventional education plus VR videos), in addition to the exact oral and written instructions, patients also used a head-mounted, 3-dimensional display (Figure 2) to watch a 6-minute VR video. When watching the video, patients would feel themselves in the virtual settings of an operating room. The VR videos provided 4 parts of information to educate patients, including instructions on bowel preparation, a to-do list before the procedure, a brief introduction to specific procedures of colonoscopy, and a to-do list after a therapeutic procedure (eg, polypectomy). The device can track head movements, and patients can familiarize themselves with the operating room and use head motion to select any part they want to learn about. Patients could leave only when they finished all parts of the video.

### Outcome Measures

The primary outcome was the quality of bowel preparation, as measured by the Boston Bowel Preparation Scale (BBPS) score (total score range, 0-9, where 0 indicates extremely unsatisfactory bowel preparation and 9 indicates complete bowel preparation), evaluated by the endoscopist during the procedure, who scored from 0 to 3 for the 3 broad regions of the colon: right, transverse, and left (the cecum and ascending colon, the hepatic and splenic flexures, and the descending colon, sigmoid colon, and rectum). If the mucosa could not be visualized because of remaining stool or food residue, this segment would receive a score of 0; a score of 3 means that the whole mucosa of the colon segment can be seen clearly without residual staining, small fragments of stool, or opaque liquid. The endoscopist (D.W.) was blinded to the group of patients.

**Figure 3.**
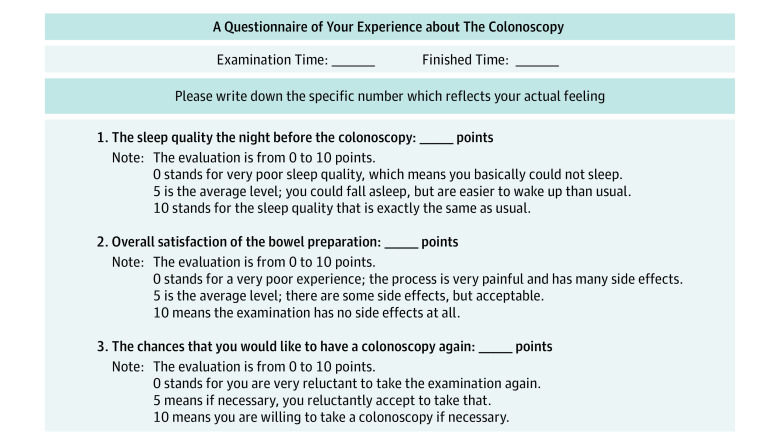
Questionnaire on Patient’s Experience of the Colonoscopy

Secondary outcomes included the rate of adequate preparation (a score ≥2 for all regions), the polyp detection rate, the biopsy-verified adenoma detection rate, and the cecal intubation rate. We used a questionnaire to evaluate patient compliance with bowel preparation (rate of complying with diet restriction and laxative use). A self-rated score from 0 to 10 points was used in the questionnaire to assess preprocedure anxiety (measured by self-rated sleep quality before the procedure [where 0 indicates very poor sleep quality and 10 indicates sleep quality that is the same as usual]), overall satisfaction with bowel preparation (where 0 indicates a very poor experience with a very painful process and many adverse effects and 10 indicates the examination has no adverse effects), and willingness to undergo another colonoscopy if indicated (where 0 indicates great reluctance to undergo the examination again and 10 indicates willingness to undergo the examination again if necessary) (Figure 3).

### Sample Size

The sample size estimation was based on the test of 2 independent proportions with a 2-sided α = .05 and a power probability of 90% (β = 0.1). The rate of adequate preparation (a score ≥2 for all regions) in the control group was 70%, and we assumed an increase of 15% for the VR video group. We calculated that at least 161 evaluable patients would be required per group for the study to achieve this power.

### Statistical Analysis

Statistical analysis was performed from November 1 to December 31, 2020. For categorical data, frequencies with percentages were presented. Quantitative data were presented using mean (SD) values or median (IQR) values. Baseline characteristics (all before randomization) included age, sex, body mass index, educational level, annual personal income, living habits (including smoking, drinking, and exercise), and medical history of comorbidity (eg, hypertension, type 2 diabetes, and bronchitis).

All data were analyzed according to the intention-to-treat approach. Occurrences of the primary and secondary end points were compared between the 2 groups. Categorical variables were analyzed using the χ^2^ test or the Fisher exact test, while continuous variables were expressed as mean (SD) values and analyzed with the *t* test. Results were presented as the difference between the 2 proportions. Analyses were performed with SPSS software, version 24.0 (IBM Corp). A 2-tailed *P* < .05 was considered statistically significant.

## Results

### Baseline Characteristics of Patients

As shown in Figure 1, a total of 346 outpatients scheduled for screening or diagnostic colonoscopy were enrolled in the trial, with 173 patients randomly assigned to each group (control group: 87 women [50.3%]; mean [SD] age, 50.5 [12.5] years; VR video group: 84 women [48.6%]; mean [SD] age, 52.6 [11.4] years). Baseline characteristics were comparable between the VR video group and control group. (Table 1). The demographic information, living habits, and the characteristics of stool of the 2 groups were almost the same. Most patients in the 2 groups had abdominal symptoms (116 [67.1%] in the control group vs 107 [61.8%] in the VR video group), and some of the participants had a change of stool, such as constipation or diarrhea. A total of 24 patients (13.9%) in the control group and 26 patients (15.0%) in the VR video group had a prior abdominal operation, which may result in changes to the intestinal anatomic structure.

**Table 1.** Baseline Characteristics of the Patients

Characteristic	Patients, No./total No. (%)		*P* value
	Control group (n = 173)	VR video group (n = 173)	
Age, mean (SD), y	50.5 (12.5)	52.6 (11.4)	.11
Sex, No. (%)			
Women	87 (50.3)	84 (48.6)	.75
Men	86 (49.7)	89 (51.4)	
BMI, mean (SD)	23.4 (3.8)	23.9 (3.5)	.20
Educational level			
Primary school	7/172 (4.1)	9/172 (5.2)	.36
Junior school	21/172 (12.2)	24/172 (14.0)	
High school	25/172 (14.5)	36/172 (20.9)	
College	90/172 (52.3)	83/172 (48.3)	
Graduate school	29/172 (16.9)	20/172 (11.6)	
Income status, ¥^a^			
≤20 000	25/162 (15.4)	29/161 (18.0)	.70
30 000 to 60 000	41/162 (25.3)	47/161 (29.2)	
70 000 to <200 000	58/162 (35.8)	50/161 (31.1)	
≥200 000	38/162 (23.5)	35/161 (21.7)	
Abdominal symptoms	116 (67.1)	107 (61.8)	.31
Alcohol consumption			
Current drinking	30/169 (17.8)	30/169 (17.8)	.89
Have stopped drinking	22/169 (13.0)	25/169 (14.8)	
Never	117/169 (69.2)	114/169 (67.5)	
Cigarette smoking			
Current	24/171 (14.0)	35/172 (20.3)	.26
Past	27/171 (15.8)	22/172 (12.8)	
Never	120/171 (70.2)	115/172 (66.9)	
Exercise, No. (%)			
Usually	71 (41.0)	55 (31.8)	.20
Sometimes	87 (50.3)	102 (59.0)	
Never	15 (8.7)	16 (9.2)	
Comorbidities, No. (%)			
Hypertension	33 (19.1)	43 (24.9)	.19
Type 2 diabetes	10 (5.8)	16 (9.2)	.22
Bronchitis	10 (5.8)	15 (8.7)	.30
No other diseases	106 (61.3)	90 (52.0)	.08
Long-term use of laxative	48 (27.7)	51 (29.5)	.72
Constipation	42 (24.3)	33 (19.1)	.25
Diarrhea	35 (20.2)	33 (19.1)	.77
Mucosanguineous feces	42 (24.3)	36 (20.8)	.42
History of abdominal operation	24 (13.9)	26 (15.0)	.78

Abbreviations: BMI, body mass index (calculated as weight in kilograms divided by height in meters squared); VR, virtual reality.

^a^ The current exchange rate of $1 to ¥6.39 was used.

#### Primary Outcome

The mean (SD) BBPS score was significantly lower in the control group compared with the VR video group (7.04 [1.70] vs 7.61 [1.65]; *P* = .002) (Table 2). Mean (SD) BBPS scores for the ascending and descending colon were also significantly higher in the VR video group than in the control group (2.29 [0.78] vs 1.97 [0.81] for the ascending colon; *P* < .001; 2.66 [0.54] vs 2.45 [0.62] for the descending colon; *P* = .001). For the transverse colon, the difference in mean (SD) scores between the 2 groups was not statistically significant (2.67 [0.60] in the control group vs 2.78 [0.51] in the VR video group; *P* = .09).

**Table 2.** Outcomes of the Colonoscopic Procedure

Outcome	Patients, No. (%)		*P* value
	Control group (n = 173)	VR video group (n = 173)	
BBPS score, mean (SD)	7.04 (1.70)	7.61 (1.65)	.002
Ascending	1.97 (0.81)	2.29 (0.78)	<.001
Transverse	2.67 (0.60)	2.78 (0.51)	.09
Descending	2.45 (0.62)	2.66 (0.54)	.001
Adequate bowel preparation	125 (72.3)	139 (80.3)	.08
Polyp detection rate	46/172 (26.7)	72/172 (41.9)	.003
Adenoma detection rate	38/172 (22.1)	56/172 (32.6)	.03
Cancer detection rate	5 (2.9)	5 (2.9)	>.99
Serrated adenoma	1 (0.6)	1 (0.6)	>.99
Cecal intubation rate	161/171 (94.2)	159/170 (93.5)	.81
Withdraw time, mean (SD), min	7.57 (1.42)	7.84 (1.46)	.09
Compliance	87 (50.3)	119 (68.8)	<.001
Overall satisfaction, mean (SD), score	8.16 (2.15)	8.68 (1.70)	.01
Sleep quality, mean (SD), score	7.08 (2.64)	7.60 (2.20)	.04
Willingness to take another colonoscopy, mean (SD), score	8.29 (2.44)	8.69 (2.34)	.12

Abbreviations: BBPS, Boston Bowel Preparation Scale; VR, virtual reality.

#### Secondary Outcomes

The rate of adequate bowel preparation was higher in the VR video group than in the control group (139 [80.3%] vs 125 [72.3%]), although the difference did not reach statistical significance (Table 2). In total, 118 polyps were detected in the study, among which 94 were adenomas. Five cancers were also found, and 1 serrated adenoma was diagnosed by colonoscopy in each group. The detection rates of polyps (72 of 172 [41.9%] vs 46 of 172 [26.7%]; *P* = .003) and adenomas (56 of 172 [32.6%] vs 38 of 172 [22.1%]; *P* = .03) were both higher in the VR video group. Cancer and serrated adenoma detection rates were comparable between the control group and the VR video group (cancer detection rate, 5 [2.9%] vs 5 [2.9%]; serrated adenoma detection rate, 1 [0.6%] vs 1 [0.6%]). The cecal intubation rate was 94.2% in the control group (161 of 171) vs 93.5% in the VR video group (159 of 170) (*P* = .81). The mean (SD) withdraw times were comparable between the control group and the VR video group (7.6 [1.4] minutes vs 7.8 [1.5] minutes; *P* = .09; Table 2). The polyp detection rate was 34.9% (112 of 321) for patients with a BBPS score of 5 or higher compared with 21.7% (5 of 23) for patients with a BBPS score of less than 5 (*P* = .20).

### Patient Experience

All participants finished the questionnaire. Mean (SD) overall satisfaction scores (8.68 [1.70] vs 8.16 [2.15]; *P* = .01) and sleep quality scores (7.60 [2.20] vs 7.08 [2.64]; *P* = .04) were significantly higher in the VR video group compared with the control group (Table 2). Patients in the VR video group had better compliance than those in the control group (119 [68.8%] vs 87 [50.3%]; *P* < .001). The mean (SD) score for willingness to accept a second colonoscopy was not statistically significantly different between the VR video group and the control group (8.69 [2.34] vs 8.29 [2.44]; *P* = .12).

## Discussion

Bowel preparation is a critical factor associated with the quality of a colonoscopy.^17^ The effect of colonoscopy screening and surveillance depends on adequate visualization and resection of polyps and adenoma, which rely on the quality of bowel cleansing.^18^ Current guidelines also recommend early repeated colonoscopic procedures when bowel preparation quality is inadequate (any colon segment BBPS score <2).^4,19^ Patients’ personal characteristics, including age, sex, body mass index, educational level, annual income, complications, and waiting time, can affect the quality of bowel preparation.^7,8,20,21^ Thus, education becomes an important means to motivate patients and improve compliance for colonoscopy preparation. Studies have demonstrated the effectiveness of various patient educational methods to enhance bowel preparation.^9,10,11,22^

New techniques have been invented and implemented into the health care industry; for instance, artificial intelligence has been implemented in image identification and disease diagnosis.^23^ Virtual reality is a new, emerging technology that can provide users with a sense of immersion. Apart from its widespread application in entertainment, VR has also been introduced into medicine and is primarily used in anatomy education, endoscopist and surgeon training, and surgical plan formulation.^24,25^ Virtual reality has also been adopted to treat attention-deficit/hyperactivity disorder and relieve pain and anxiety by distracting attention.^26,27,28^ In some medical centers, VR was used to replace procedural sedation and analgesia during colonoscopic procedures to ease pain, and a survey found that about one-fourth of respondents are willing to choose VR first.^29^ Another study reported that the use of VR glasses during a colonoscopy was acceptable to patients and did not compromise the technical success of the endoscopic procedure. Moreover, no differences have been found between VR and procedural sedation and analgesia in comfort, pain, anxiety, and satisfaction.^30^ Hence, we intended to explore the possibility of extending the application of VR to deliver precolonoscopy patient education.

In our study, the BBPS score of the control group was lower than that of the VR video group, which shows that the application of VR can significantly improve bowel preparation quality. The VR video intervention was also associated with higher polyp and adenoma detection rates, reflecting adequate bowel preparation. This finding is consistent with previous studies that found that new educational methods could improve polyp and adenoma detection rates.^9,10,12^ In addition, the rate of adequate preparation in the VR video group was higher than that in the control group, although it did not reach statistical significance. The difference can be partly explained by the comparatively higher educational level and socioeconomic status among patients in the VR video group, which resulted in higher baseline bowel preparation scores.

We also found that using VR videos enhanced patients’ compliance and experience, resulting in the improvement of bowel preparation. This finding can be explained by the following reasons. First, compared with conventional education methods, this novel technology can arouse patients’ interest and motivation to explore what they need to pay attention to before and after the procedure. The sense of reality established by VR can also help patients understand and memorize what to do during bowel preparation. Second, it is believed that VR videos can make patients focus on the announcements, avoiding distraction from the surroundings, which can further improve the effectiveness of patient education.^31^

Patients’ willingness to undergo a colonoscopy and their pretest anxiety were other outcomes of this study. Patient anxiety before an invasive procedure is common. Severe anxiety may even lead to withdrawal from a colonoscopy and increase the risk of adverse advents.^32^ Yang et al^33^ concluded that patients who underwent a colonoscopy had a higher level of anxiety, especially women and those with a history of functional abdominal pain, a lower educational level, and a lower income level. Patents worried about bowel preparation, embarrassment, pain, possible complications of the procedure, and possible serious diagnosis.^34^ Our findings further support the idea that when patients obtain information about the procedure in an easy way before the colonoscopy, their anxiety could be effectively reduced.^35^ Another study found that VR videos could improve patient sleep quality the night before a colonoscopic examination, supporting its efficacy in reducing patient anxiety.^36^ Medical information conveyed in a vivid and attractive way by VR videos can reduce anxiety.^37,38^

### Limitations

There are several limitations of this study. First, the trial was a single-center study conducted in a tertiary hospital in Beijing, China. The educational level and economic status of the patients were usually above the mean level of the general population, which may limit the generalizability of our findings. Second, we used self-rated sleep quality to measure the level of anxiety, rather than using other scientific scales (such as the State-Trait Anxiety Inventory). Because only outpatients were enrolled in this study, it was difficult to request that patients complete a complicated psychological scale during the busy outpatient service. However, previous research has found that sleep quality can represent the patient’s level of anxiety.^36,39^ Although the polyp detection rate was higher for patients with better bowel preparation (BBPS score ≥5), such a difference did not reach statistical significance. This may be because the polyp detection rate was not the primary end point of our study, and the trial was not powered to demonstrate the correlation between grades of bowel preparation and the polyp detection rate. Finally, several previous studies have also demonstrated that telephone calls or message reminders could improve the quality of bowel preparation.^12,13,22^ Nevertheless, we only compared the effect of VR videos in patient education with conventional methods of education. It would be helpful in future studies to clarify whether another unique patient education method or a reminder before appointments is equally efficacious.

## Conclusions

This randomized clinical trial has found that using VR videos for patient education could improve the quality of bowel preparation and increase the rate of detection of polyps and adenomas. Moreover, it could reduce patient anxiety before a colonoscopy and increase patient satisfaction.

## Notes

Supplement 1.
Trial Protocol

Supplement 2. **eFigure.** The Written Instruction Containing Food and Laxatives Details

Supplement 3.
Data Sharing Statement
