# Supplementary material for: Educating Outpatients for Bowel Preparation Before Colonoscopy Using Conventional Methods vs Virtual Reality Videos Plus Conventional Methods: A Randomized Clinical Trial
Source: JAMA Netw Open. 2021 Nov 22;4(11):e2135576. doi: 10.1001/jamanetworkopen.2021.35576 (PMC8609410; doi:10.1001/jamanetworkopen.2021.35576)
Supplement: Supplement 1. — Trial Protocol [file jamanetwopen-e2135576-s001.pdf]

Educational Virtual Reality Videos in Improving Bowel Preparation Quality and Satisfaction of Outpatients Undergoing Colonoscopy: Protocol of A Randomized Controlled Trial

Design

The trial is a prospective, randomized, controlled, single-blinded, single-center trial. Outpatients arranged to undergo a conscious colonoscopy (i.e., without sedation) for screening or diagnostic purposes for the first time will be randomized to the control group or the VR intervention group. This study, compared with conventional patient education methods, aims to explore whether VR videos can improve the bowel preparation quality, increase patient adherence and satisfaction, and reduce pre-procedure anxiety.

Study Population

All patients who have the indications for colonoscopy screening presenting to Peking Union Medical College Hospital, a tertiary hospital in Beijing, China, will be assessed for eligibility during the appointment.

Inclusion criteria

- Outpatients indicated for elective colonoscopy: 1) For screening purposes: asymptomatic patients with average or high risk for colorectal cancer; 2) For diagnostic purposes: patients presented with abnormal imaging or lower gastrointestinal symptoms including bloody stool, chronic diarrhea and abdominal pain
- No prior colonoscopy
- Age 18-75 years
- Written informed consent

Exclusion criteria

Patient who meets any of the following criteria will be excluded:

- History of bowel surgery
- Diagnosed with severe comorbidities (e.g., ascites, congestive heart failure, chronic renal failure, coronary artery disease within the last six months)
- On constipation, laxatives, or anti-diarrheal medications
- Pregnant
- Severe constipation (<3 bowel movement/week)
- Inflammatory bowel disease (IBD)
- Contradictions for colonoscopy
- Unable to watch VR videos (e.g., blindness)

Randomization

After the colonoscopy is scheduled and written informed consent obtained, patients will be randomized with 1:1 ratio to the coventional education method or the conventional education plus VR video. The randomization process will be run using the R software (open source, www.r-project.org) in random blocks of four.

General bowel preparation requirement

Diet restriction: Low-residue diet until the evening on the day before colonoscopy

Colon cleansing regimens:

The first dose: 2L laxatives (polyethylene glycol) used on the evening of the day before colonoscopy (after dinner)

The second dose: 1L laxatives used 3-4 hours before colonoscopy

The control group: conventional patient education methods

Patients in the control group will only receive routine patient education on bowel preparation of colonoscopy. A well-trained nurse or a doctor will provide oral instructions on bowel preparation (including definition, significance, correct steps as well as dietary limitations). Written instructions are also provided to patients to take away, which have the same contents as the oral instructions.

The intervention group: conventional methods plus VR videos

In addition to the routine patient education methods mentioned above, patients in the intervention group will watch a VR video for about 6 minutes. Videos will give instructions on bowel preparation step by step, emphasize on points for attention before and after the procedure, and give brief introductions to the procedures of colonoscopy and a to-do list after a therapeutic procedure (e.g., polypectomy).

Primary endpoint

The primary endpoint is the quality of bowel preparation measured by the Boston bowel preparation score evaluated during the procedure. Endoscopists are blinded to the grouping of patients.

Secondary endpoints

We also hypothesize that VR videos can increase patient motivation and deepen their understanding of colonoscopy, which is likely to increase the detection rate of abnormity, reduce anxiety, and improve patient experiences. We set secondary endpoints as follows:

- Polyp detection rate (PDR).
- Adenoma detection rate (ADR).
- Cecal intubation rate.
- Patient compliance with bowel preparation (diet restriction and laxatives use).
- Withdrawal time.
- Pre-procedure anxiety (measured by self-rated sleep quality before the procedure).

- Overall satisfaction with bowel preparation.
- Willingness to take another colonoscopy if indicated.

Sample size calculation

The sample size estimation was based on the test of 2 independent proportions with a 2-sided $\alpha=0.05$ and a power probability of 90% ($\beta=0.1$). The rate of adequate preparation (a score≥2 for all regions) in the control group is 70%[3], and we assumed an increase of 15% for the VR Group. We calculated that at least 161 evaluable patients would be required per group for the study to achieve this power.

Data collection and follow-up

Data collection will be performed by using a standardized case report form. DSMC will verify all primary and secondary endpoints as well as at least 10% of data in case report forms against on-site source data. Discrepancies detected by the committee will be resolved through a consensus by two investigators unaware of the study group assignment and not involved in patient care.

Descriptive statistics

For categorical data, frequencies will be presented. Quantitative data will be presented as the mean and standard deviation or median and interquartile range. Baseline characteristics (all prior to randomization) are: age, sex, body mass index, education level, annual personal income, living habits (including smoking, drinking and exercise), dietary habit (vegetarianism/meatatarian/balanced diet), past medical history of comorbidity (hypertension, diabetes mellitus, bronchitis, asthma, congestive heart failure, chronic renal failure, coronary artery disease, IBD, malignancy), symptoms (chronic diarrhea, constipation, mucous stool or bloody stool), family history of colorectal cancer or specific inherited syndromes.

Analyses

All data will be analyzed according to the intention-to-treat approach in which all randomized patients are included. Occurrences of the primary and secondary endpoints are compared between the two groups. Results are presented as risk ratios with corresponding 95% confidence intervals. A two-tailed $P< 0.05$ is considered statistically significant.

Trial Management

A steering committee will manage the trial. Screening and recruitment will be reviewed at monthly meetings. An independent data and safety monitoring committee (DSMC) will meet regularly to ensure patient safety and data quality. After the colonoscopy of the last patient in the trial has been performed, an adjudication

committee blinded for the treatment allocation will evaluate each patient using the raw data. Disagreements will be resolved in a plenary consensus meeting. Relevant clinical and radiological data submitted to the steering committee will facilitate duplicate blinded outcome adjudication.

Termination of the trial

An interim analysis will be conducted on the primary endpoint when 25%, 50%, and 75% of patients have been enrolled. The interim analysis is performed by an independent statistician, blinded for the treatment allocation. The statistician will report to the independent DSMC. The DSMC will have unrestricted access to all data and will discuss the results of the intention-to-treat analysis with the steering committee in a joint meeting. The steering committee decides on the continuation of the trial and will report to the central ethics committee. The Peto approach is used to terminate the trial when the intervention group has a significant benefit from the addition of VR to the patient education methods using symmetric stopping boundaries at $P<0.001$. The trial will not be stopped in case of futility unless the DSMC during safety monitoring advises otherwise. In this case, DSMC will discuss potential stopping for futility with the trial steering committee.

Safety

The DSMC will monitor the progress of the trial by examining safety variables quarterly. This evaluation is based on unblinded data, in the presence of the study coordinator when DSMC requires details of the study. After the full explanation of the data is presented, the study coordinator is dismissed, and the DSMC discusses the consequences of the data presented. Adverse events are defined as "any undesirable experience occurring to a subject during a clinical trial, whether or not considered related to the intervention," such as a sense of dizziness after watching VR videos. All participating physicians will be asked to report any potential adverse events. These adverse events will be listed and discussed with the DSMC. The outcome of the meeting of the DSMC will be discussed with the trial steering committee. The outcome will also be sent to our hospital institutional review board (IRB). The DSMC will evaluate the data of the deceased patients for the cause of death, and possible trial related severe adverse events.
