# Supplementary material for: Educating Outpatients for Bowel Preparation Before Colonoscopy Using Conventional Methods vs Virtual Reality Videos Plus Conventional Methods: A Randomized Clinical Trial
Source: JAMA Netw Open. 2021 Nov 22;4(11):e2135576. doi: 10.1001/jamanetworkopen.2021.35576 (PMC8609410; doi:10.1001/jamanetworkopen.2021.35576)
Supplement: Supplement 2. — eFigure. The Written Instruction Containing Food and Laxatives Details [file jamanetwopen-e2135576-s002.pdf]

Supplementary Online Content

Chen G, Zhao Y, Xie F, et al. Educating outpatients for bowel preparation before colonoscopy using conventional methods vs virtual reality videos plus conventional methods: a randomized clinical trial. *JAMA Netw Open.* 2021;4(11):e2135576. doi:10.1001/jamanetworkopen.2021.35576

eFigure. The Written Instruction Containing Food and Laxatives Details

This supplementary material has been provided by the authors to give readers additional information about their work.

© 2021 Chen G et al. *JAMA Network Open.*

eFigure. The Written Instruction Containing Food and Laxatives Details. The instruction was given to all patients on the same day they accepted oral education and watched VR videos.

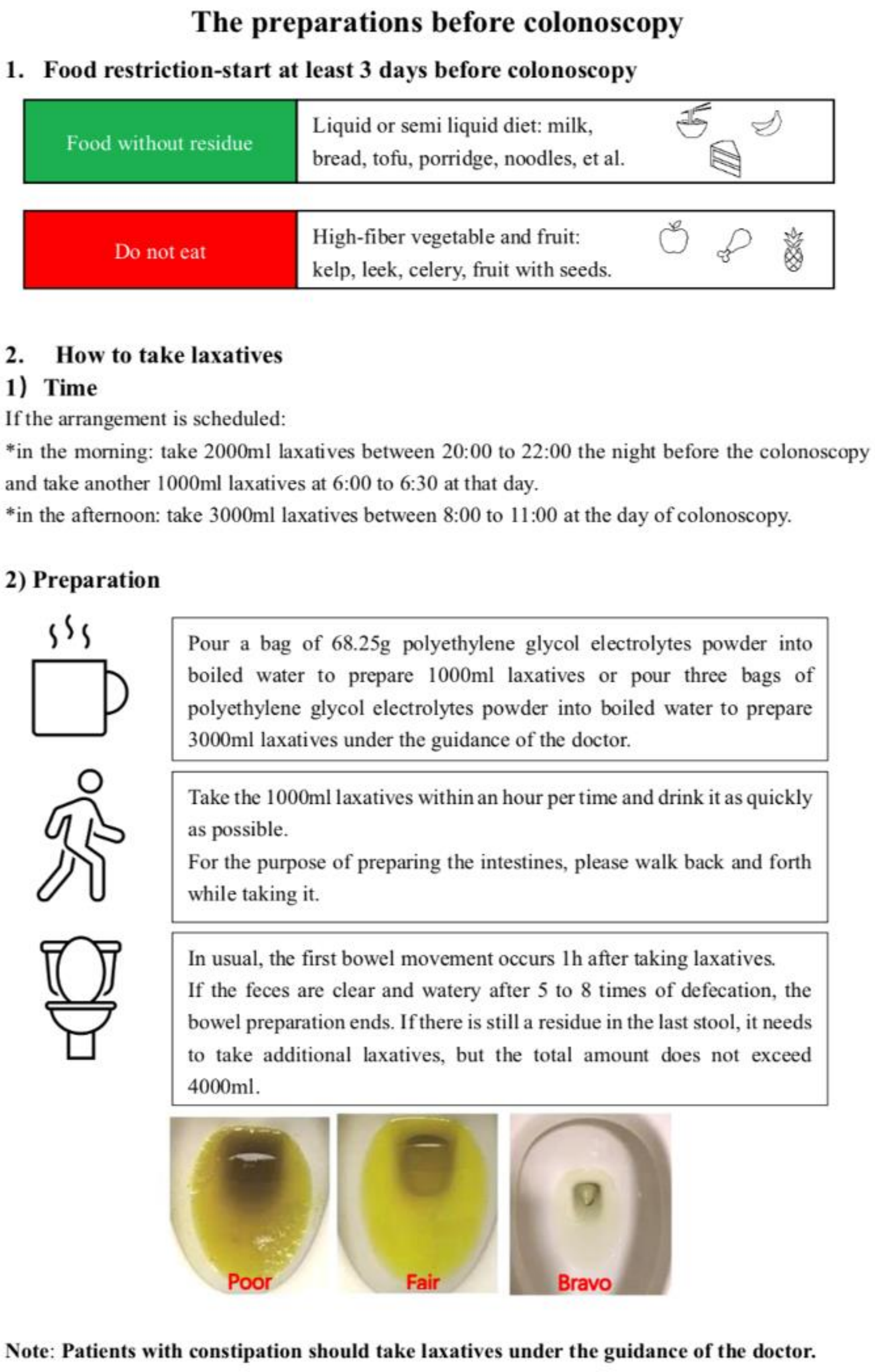

The preparations before colonoscopy

1. Food restriction-start at least 3 days before colonoscopy

Food without residue	Liquid or semi liquid diet: milk, bread, tofu, porridge, noodles, et al.
Do not eat	High-fiber vegetable and fruit: kelp, leek, celery, fruit with seeds.

2. How to take laxatives

1) Time

If the arrangement is scheduled:

*in the morning: take 2000ml laxatives between 20:00 to 22:00 the night before the colonoscopy and take another 1000ml laxatives at 6:00 to 6:30 at that day.

*in the afternoon: take 3000ml laxatives between 8:00 to 11:00 at the day of colonoscopy.

2) Preparation

Pour a bag of 68.25g polyethylene glycol electrolytes powder into boiled water to prepare 1000ml laxatives or pour three bags of polyethylene glycol electrolytes powder into boiled water to prepare 3000ml laxatives under the guidance of the doctor.

Take the 1000ml laxatives within an hour per time and drink it as quickly as possible.
For the purpose of preparing the intestines, please walk back and forth while taking it.

In usual, the first bowel movement occurs 1h after taking laxatives.
If the feces are clear and watery after 5 to 8 times of defecation, the bowel preparation ends. If there is still a residue in the last stool, it needs to take additional laxatives, but the total amount does not exceed 4000ml.

Poor Fair Bravo

Note: Patients with constipation should take laxatives under the guidance of the doctor.

© 2021 Chen G et al. *JAMA Network Open.*
