# Supplementary material for: Educating Outpatients for Bowel Preparation Before Colonoscopy Using Conventional Methods vs Virtual Reality Videos Plus Conventional Methods: A Randomized Clinical Trial
Source: JAMA Netw Open. 2021 Nov 22;4(11):e2135576. doi: 10.1001/jamanetworkopen.2021.35576 (PMC8609410; doi:10.1001/jamanetworkopen.2021.35576)
Supplement: Supplement 3. — Data Sharing Statement [file jamanetwopen-e2135576-s003.pdf]

Data Sharing Statement

Chen. Educating Outpatients for Bowel Preparation Before Colonoscopy Using Conventional Methods vs Virtual Reality Videos Plus Conventional Methods. *JAMA Netw Open*. Published November 22, 2021. doi:10.1001/jamanetworkopen.2021.35576

Data
Data available: No
